# Living donor liver transplantation with two-stage hepatectomy for patients with isolated, irresectable colorectal liver—the LIVER-T(W)O-HEAL study

**DOI:** 10.1186/s12957-018-1549-5

**Published:** 2019-01-08

**Authors:** Falk Rauchfuß, Silvio Nadalin, Alfred Königsrainer, Utz Settmacher

**Affiliations:** 1 Department of General, Visceral and Vascular Surgery, Jena University Hospital, 07747 Jena, Germany; 2 Department of General, Visceral and Transplant Surgery, University Hospital Tübingen, Tübingen, Germany

**Keywords:** Colorectal cancer, Living donor liver transplantation, Hepatectomy, Liver resection, ALPPS procedure, Portal vein ligation

## Abstract

**Background:**

Colorectal cancer is the third most common malignancy worldwide. The occurrence of liver metastases worsens the prognosis of the patient significantly if the tumor burden is not resectable.

Liver transplantation might be an option for otherwise irresectable colorectal liver metastases. In this study, we evaluate the role of two-stage hepatectomy in combination with a left-lateral living donor liver transplantation.

**Methods:**

Patients with irresectable liver metastases having a stable disease or tumor regression after at least 8 weeks of systemic chemotherapy without an extrahepatic tumor burden (except resectable lung metastases) are suitable for study inclusion. A randomization is not planned since the control arm (systemic chemotherapy) is well established and the superiority of the transplantation procedure has to be expected.

The surgical treatment consists of two steps: in a first operation, a left hemihepatectomy in the recipient will be performed. At this place, the left lateral liver lobe (segments II and III) of a living donor will be transplanted. To induce a growth of the graft, a portal vein ligation will be performed. Approximately after 2 weeks, the removal of the right hemiliver will be conducted if the control imaging shows a sufficient growth of the graft.

**Results:**

The patient recruitment is ongoing. In total, three patients have been already transplanted with this protocol. Up to now, they are tumor-free and in good clinical health.

**Discussion:**

With the design of the LIVER-T(W)O-HEAL study, it might be possible to offer patients with otherwise irresectable colorectal liver metastases a curative treatment option. The key point of this study will be, most probably, the patient’s selection.

**Trial registration:**

Registered at Clinical Trials; NCT03488953; registered on April 5, 2018

**Electronic supplementary material:**

The online version of this article (10.1186/s12957-018-1549-5) contains supplementary material, which is available to authorized users.

## Background

Colorectal cancer (CRC) is the third most common malignancy worldwide [1]. Approximately 50% of patients suffering from colorectal cancer have developed or will develop liver metastases (CRLM) [2]. Nowadays, systemic chemotherapeutic agents (alone or in combination with antibodies) can provide for patients with irresectable CRLM (i-CRLM) a median survival time of nearly 23 months and a 5-year survival rate in the metastatic situation around 20% [3].

The achievement of a R0 situation significantly improves the outcome since the 5-year survival rate in patients who had a curative liver resection is > 50% [4, 5]. Despite recent advantages in hepatobiliary surgery (introduction of the ALPPS procedure [6], combination of liver resection and ablative therapies [7], conditioning of the remaining liver parenchyma [8]), only 20–30% of patients with liver metastases are eligible for curative liver resection at the time of diagnosis [9].

The mechanistic approach to reach a R0 situation performing a complete hepatectomy followed by liver transplantation for otherwise irresectable liver metastases was already considered in the early 1990s. However, the largest series by Mühlbacher et al. reported a 5-year survival rate of only 12% [10]. Due to the organ shortage in most countries with mainly deceased donation programs and the bad results reported in the literature, liver transplantation for colorectal liver metastases was abandoned. In fact, especially Gorgen et al., but also Moris et al., proposed a subdivision in two different eras: one having performed liver transplantation for CRLM prior the year 2000 (having bad results) and the other after 2000 (with much better results, most probably due to an improved patient’s selection) [11, 12].

Especially the recent publications from Norway showed 5-year survival rates up to 60%. Actually, there are five trials recruiting patients for liver transplantation (Table 1).

**Table 1:** Five trials recruiting patients with colorectal liver metastases for liver transplantation

	RAPID	SECA-II			LIVER-T(W)O-HEAL
	NCT02215889	NCT01479608	NCT02597348	NCT02864485	NCT03488953
Country	Norway	Norway	France	Canada	Germany
Center(s)	Oslo	Oslo	Paris, Paul-Brousse- Hospital	Toronto	Jena and Tübingen
Start	6/2014	2011	10/2015	8/2016	3/2018
State	Recruiting	Recruiting	Recruiting	Recruiting	Recruiting
Planned number of included patients	20	25	90	20	40
Inclusion criteria	More than 8 weeks of systemic therapy More than 12 months after resection of the primary tumor.	More than 6 weeks of systemic therapy More than 12 months after resection of the primary tumor. Metachronous metastasis > 12 months after resection of the primary tumor Initial lymph node state: pN0 CEA: < 100	At least “stable disease” after 3 months of systemic therapy. More than 12 months after resection of the primary tumor. BRAF wild-type	At least “stable disease” during systemic therapy. State of the primary tumor: <pT3, N1 More than 6 months after resection of the primary tumor. BRAF wild-type	At least “stable disease” after 8 weeks of systemic therapy. State of the primary tumor: <pT3, N1
Mode of transplantation	Deceased donation. Left lateral split (segment II/III). Two-stage hepatectomy.	Deceased donation, whole organ	Deceased donation, whole organ	Living donation, right liver lobe	Living donation Left lateral split (segment II/III) Two-stage hepatectomy
Primary endpoint	Overall survival, recurrence-free survival, technical feasibility.	Overall survival, recurrence-free survival	Overall survival, recurrence-free survival	Overall survival, recurrence-free survival	Overall survival, recurrence-free survival, technical feasibility

In Germany, colorectal cancer represents the third most common malignancy with the aforementioned remarks in case of a metastatic disease [13]. However, CRLM are not an accepted indication in the German guidelines for liver transplantation. Furthermore, the organ shortage in Germany has hit an all-time low in 2017 with 9.3 deceased organ donors per one million inhabitants [14]. Therefore, every new indication for liver transplantation using deceased donations has to be considered carefully.

Concluding the aforementioned data, we developed a clinical study offering well-selected patients with irresectable colorectal liver metastases the possibility for liver transplantation in Germany. Using living donors, we do not draw on the restricted pool of deceased organs. We have chosen the mode of liver resection with the minimal loss of liver parenchyma in the donor. The concept of the study resembles with the protocol of the Norwegian RAPID study [15] with the exception that a left lateral lobe of a living donor is used for the transplantation procedure as recently reported by Königsrainer et al. [16].

Thus, we introduce the LIVER-T(W)O-HEAL protocol, where a two-stage hepatectomy after left lateral living donor liver transplantation is performed.

## Methods/design

### Purpose

The purpose of the present study is to evaluate a two-stage hepatectomy with a left lateral living donor liver transplantation and (subtotal) right portal vein ligation for treatment of otherwise irresectable liver metastases of colorectal carcinoma in curative intent.

### Study setting

**Fig. 1:**
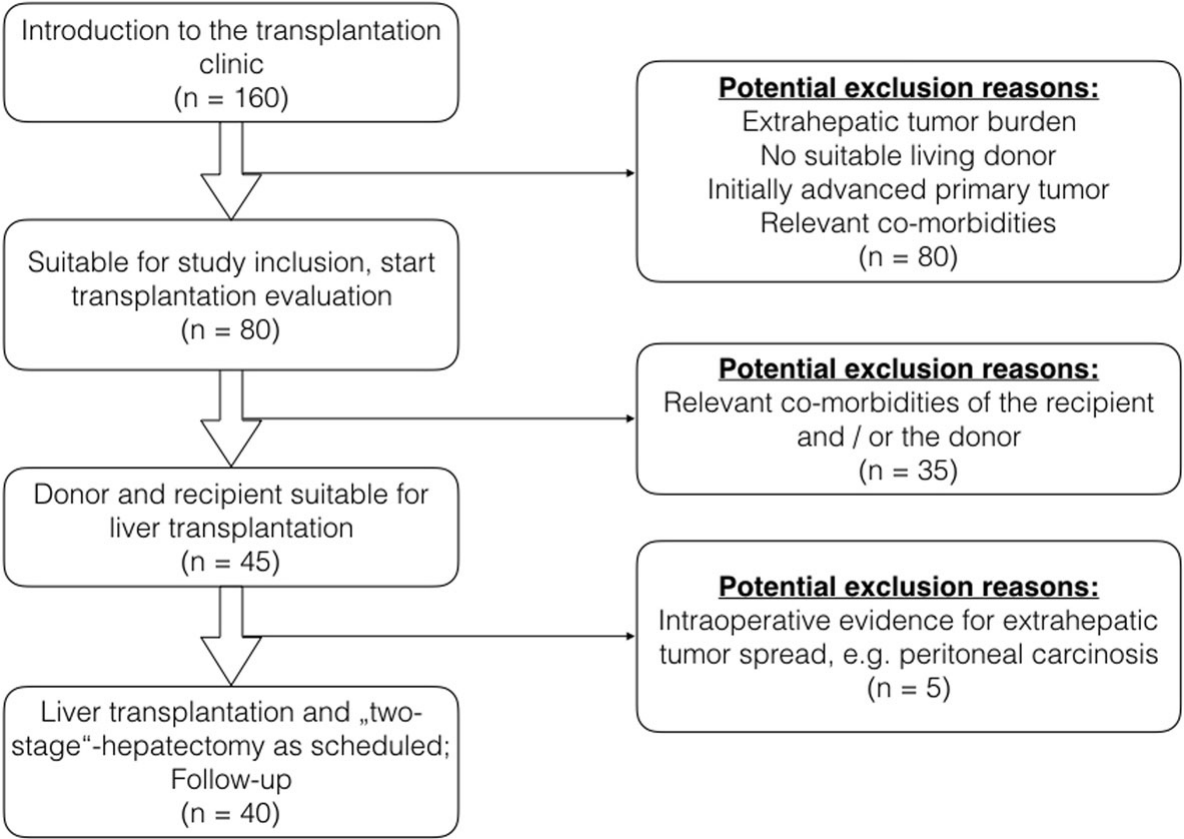
Estimated patient flow in the LIVER-T(W)O-HEAL-study

The study is an investigator-initiated, bi-institutional, one-arm trial. The potential patient flow of the trial is shown in Fig. 1.

For ethical reasons, we decided to perform no control group since the superiority of the liver transplantation procedure is to be expected. Therefore, we decided to compare our transplantation cohort with a historic control group of patients with i-CRLM, who has undergone the actual gold standards of chemotherapy.

A Standard Protocol Items: Recommendations for Interventional Trials (SPIRIT) checklist is provided in Additional file 1.

### Endpoints

#### Primary endpoint

The primary endpoint is the overall patient survival 36 months after the second step of living donor liver transplantation in a two-stage procedure. This time point has been chosen since the patient has to be regarded as “free of tumor” from this last step of tumor operation.

#### Secondary endpoints

Secondary endpoints are:

- The recurrence-free survival of the patients 36 months after the second stage of hepatectomy
- The medical and psychological morbidity of both donor and recipient, defined as complications ≥ IIIb according to the Clavien-Dindo classification [17] and as proposed by Nadalin et al. [18]

### Patient selection

All patients with irresectable colorectal liver metastases (whereby the irresectability is evaluated by an experienced, independent hepatobiliary surgeon) and no extrahepatic tumor burden (except resectable lung metastases) are potential candidates for study inclusion, if:

- The tumor burden is at least a “stable disease,” according to the RECIST criteria [19], after a minimum of 8 weeks of systemic chemotherapy
- An external, independent review board, composed of a surgeon, an oncologist, and a radiologist, checked and approved the criteria for study inclusion

### Exclusion criteria

Patients are ineligible for study participation, if:

- There is an extrahepatic tumor burden (except resectable lung metastases) and/or a macroscopic vascular tumor infiltration
- No suitable donor available
- Significant comorbidities that preclude transplantation
- There is a tumor progression during chemotherapy

### Treatment methods

The time schedule for both, donor and recipient, is displayed in Table 2.

**Table 2:** Time schedule for both donor and recipientᅟ

	Introduction transplantation clinic	Recipient evaluation	Donor evaluation	Operation, step 1	Days 1–7 after step 1	Days 8, 10, 14, 21 after step 1	Discharge from the hospital, donor	Day prior step 2	Operation, step 2	Days 1–7 after step 2	Days 8, 10, 14, 21 after step 2	Discharge from the hospital, recipient	3 months after step 2	6 months after step 2	9 months after step 2	12 months after step 2	18 months after step 2	24 months after step 2	30 months after step 2	36 months after step 2 —close out- visit
Anamnesis	X		X																	
Written consent donor and recipient			X																	
In- and exclusion criteria	X	X	X																	
Physical examination	X	X	X																	
Acquisition of ongoing immunosuppression					X	X		X	X	X	X	X	X	X	X	X	X	X	X	X
Laboratory analysis		X	X	X	X	X	X	X	X	X	X	X	X	X	X	X	X	X	X	X
Adverse events				X	X	X	X	X	X	X	X	X	X	X	X	X	X	X	X	X
Computed tomography of chest and abdomen		X	X					X						X	X		X		X	
MeVis-analysis			X	X				X					X	X						
MRCP			X																	
PET-CT		X											X			X		X		X
Acquisition of adjuvant chemotherapy													X	X	X	X	X	X	X	X
LiMax test (voluntary)		X	X					X				X	X			X		X		X
Functional MR-scan			X					X					X							

If the potential recipient fulfills the in- and exclusion criteria and has a potential suitable living donor, the patient will be admitted to a special transplantation ward, where the evaluation process for liver transplantation in this special setting begins. The procedure includes a FDG-PET-CT scan for tumor burden additional to the standard evaluation for liver transplantation.

If contraindications for liver transplantation are excluded, the potential recipient will be discussed in the transplantation board and subsequently listed as organ recipient at Eurotransplant.

Furthermore, the patient’s history and all available imaging will be sent to an external review board, consisting of an experienced hepatobiliary surgeon, an oncologist, and a radiologist, who will assess the individual case from their specialized field. Only if all three reviewers approve the study inclusion of the patient, the following steps are performed.

Now, the potential donor will be evaluated according a standardized multistep evaluation protocol [20]. In particular, the dataset of CT and MRI scans will be sent to MeVis (Bremen) for a 3D virtual case analysis of vascular and biliary anatomy as well exact computing of liver volumetry. Furthermore, the evaluation process includes a cardiologic examination and the premedication visit as well as a LiMAx test. Both, donor and recipient, will be interviewed by a clinical psychologist.

At the end of the evaluation procedure, the individual patient case will be judged by an independent living donation committee of the respective State Chamber of Physicians.

#### Operative procedure—step 1

**Fig. 2:**
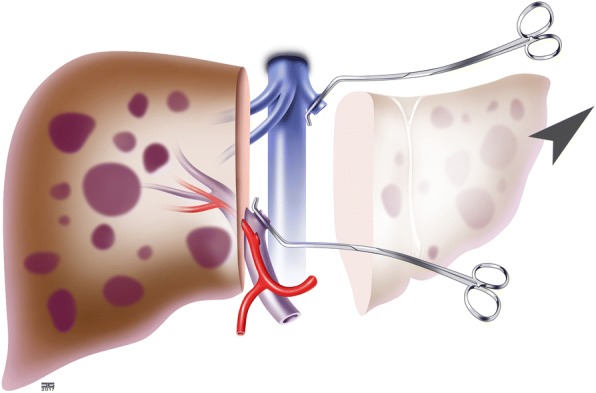
Step 1: Left hemihepatectomy in the recipient. The resection plane might vary according to the localization of the central metastases in each patient

The transplantation procedure starts with an extensive exploration of the abdominal cavity of the recipient to exclude an extrahepatic tumor manifestation. If there is no extrahepatic tumor burden, a left hemihepatectomy is performed, whereby the resection plane depends on the localization of the metastases and might vary between individual patients (Fig. 2).

Parallel, the donor procedure is started, whereby a left lateral hepatectomy (resection of the segments II and III) is performed.

**Fig. 3:**
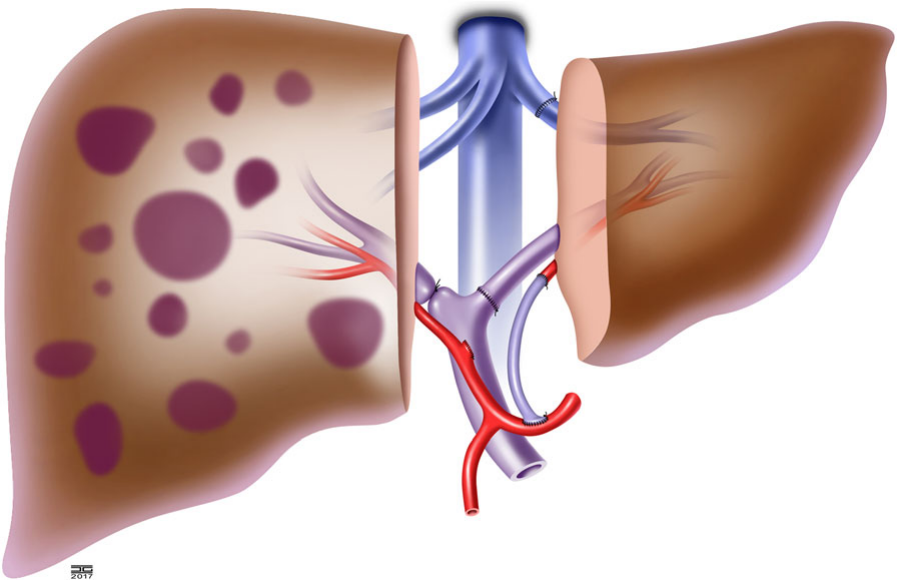
Step 1: Auxiliary transplantation of the left lateral lobe of the donor

The left lateral graft is transplanted orthotopically (Fig. 3). To induct a more rapid growth and regeneration of the graft, the right portal vein is ligated (according to the ALPPS concept) while measuring the portal pressure. Hereby, intraoperative following hemodynamic parameters will be measured at different time of the operation, and according to them a graft inflow modulation may be performed [21].

The reconstruction of the biliary tree of the graft is realized performing a bilio-digestive anastomosis. This allows an easier procurement of the right liver in step 2.

The immunosuppression is performed according to the following protocol:

**Table:** 

Intraoperative	
500 mg Methylprednisolone i.v.	
Early postoperative phase	
Tacrolimus	Target level 5–10 ng/ml
Mycophenolat-Mofetil	1000 mg twice daily
Basiliximab	20 mg i.v. on the day of the transplantation and on POD 4
Prednisolone	0.5 mg/kgBW/d from POD 1–10 and reduction of 0.1 mg/kgBW/d every 10 days
3 months after the transplantation procedure	
Everolimus in combination with Tacrolimus	Both with a target level of around 5 ng/ml

Between both operative steps, continuous laboratory analyses as well as ultrasound investigations will be performed. Presumable after 2 weeks, a CT scan, a subsequent MEVIS analysis, and a LiMAx test are performed. If a normal liver function is diagnosed, step 2 is scheduled for the following day.

#### Operative procedure—step 2

**Fig. 4:**
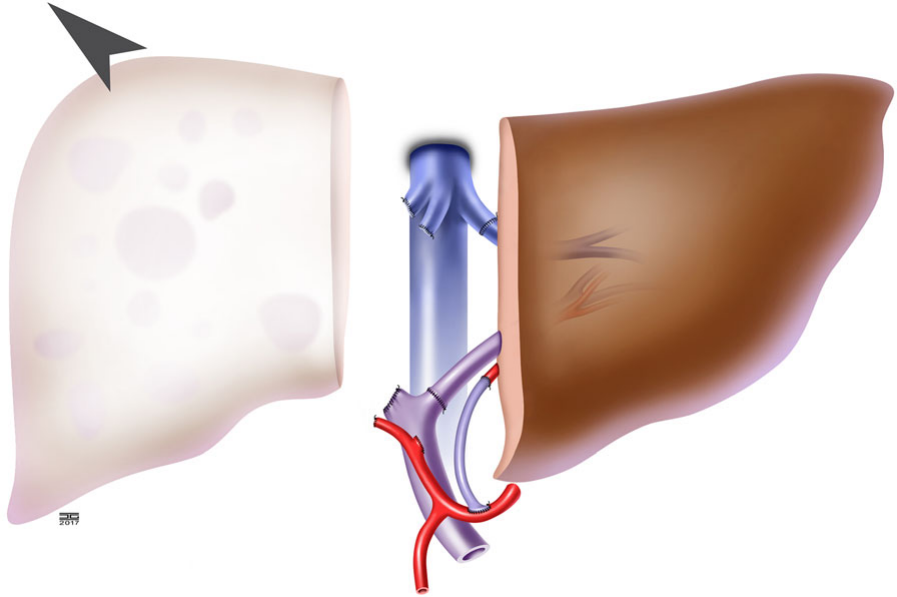
Step 2: Removal of the right hemiliver (probably 2 weeks after step 1)

In this operation, the remaining right liver will be removed (Fig. 4).

### Follow-up

The follow-up is coordinated by the transplantation unit of the centers. It includes a CT scan of thorax and abdomen 6, 9, 18, and 30 months after the finalization of the two-stage procedure (step 2). Furthermore, a PET-CT scan will be performed after 3 months and 1, 2, and 3 years after step 2. At these points in time, a LiMAx test will also be performed to assess the liver function of the graft. Furthermore, the individual immunosuppressive regime is recorded. In case of an adjuvant chemotherapy, agents, duration, and tolerance of the drug(s) will be registered.

Furthermore, CEA values and, additionally, liquid biopsies for the detection of circulating tumor DNA will be controlled.

### Statistical methods

The primary endpoint (overall survival) and the secondary endpoint “disease-free survival” are examined in a model using Gray’s test since they are competing events [22]. For both events, the calculated hazard ratios are indicated with a confidence interval of 95%.

All other secondary endpoints, reflecting the morbidity of donor or recipient, are compared using Fisher’s exact test between the groups. The absolute and relative frequency of these adverse events per group will be reported.

The significance level for all tests is defined as *α* = 0.05. The analysis is performed according to an “intention-to-treat” principle. Subgroup analysis is not planned.

### Centralized monitoring and the data and safety monitoring committee

The data monitoring will be performed by the Center of Clinical Studies in Jena and Tübingen.

### Participating institutions

The German Medical Association approved the study protocol for the liver transplantation centers in Jena and Tübingen. During this study, it is not provided to enable other transplantation centers a study participation.

## Results

The patient recruitment is ongoing. There have been already three patients transplanted in both centers.

The main reasons for study exclusion were tumor progression despite ongoing chemotherapy, the non-availability of a suitable donor, extrahepatic tumor burden (mainly lymph nodes), and logistic reasons (mainly requests from non-EU citizens who could not realize a funding for treatment in Germany).

Up to now, there was no donor morbidity or mortality. One recipient suffered from a bile leakage which was treated conservatively and recovered fully.

## Discussion

The LIVER-T(W)O-HEAL study is a prospective study which aims to assess the option of a two-stage liver transplantation for otherwise not curative treatable patients suffering from irresectable colorectal liver metastases.

The expansion of indications for liver transplantation is discussed controversially, especially in the light of an increasing organ shortage in most Western countries. Schaefer et al. did not see liver transplantation for irresectable colorectal liver metastases as standard therapy for these patients. However, despite the critical attitude of their work, the authors concede that in accordance with well-defined patient selection criteria, an expansion might be conceivable [23]. Mazzaferro et al. described liver transplantation as a potential curative-oriented option in the management of patients with colorectal liver metastases if certain conditions are fulfilled. These terms are the use of up-to-date staging protocols, restrictions on clinical conditions and tumor presentation known to affect prognosis, nonresectability has to be confirmed in centers with experience in both (liver resection and liver transplantation), sufficient life span of the transplant candidates to assess survival and life-gain achieved with liver transplantation, and priority given to the use of marginal donors. Interestingly, the authors reflected in the discussion of the last mentioned point, among others, to the use of living liver donors [24].

However, the Norwegian data showed a 100% tumor recurrence within 2 years after the transplantation. Despite this fact, the 5-year overall survival was around 60%, a result which could never be reached with the best chemotherapy regime [25]. Therefore, we are convinced that it is our duty to offer well-selected patients the possibility of a long-term survival.

The key for good long-term results, as it is also well-known for other malignant diseases, is the patient selection. Therefore, we will only accept patients as candidates for liver transplantation if there is at least a stable disease after a minimum of 8 weeks systemic chemotherapy. This is pointing at a favorable tumor biology. The next point is an exclusion of an extrahepatic tumor burden since we believe that especially an intraabdominal tumor spread (e.g., lymph node metastases or peritoneal carcinosis) is associated with a far progressed tumor disease precluding such a sophisticated approach like a liver transplantation. The only exception is resectable lung metastases since a recent published study showed that there is no difference in size dynamics of the metastases even if the patients are immunosuppressed [26].

Colorectal cancer is a common malignancy in Germany. The excellent survival rates of the Norwegian group suggest the possibility to offer this therapy option also in Germany. However, the organ shortage does not allow any expansion of the liver transplantation indications since an increasing number of patients even with established transplantation indications die on the waiting list. The Norwegian approach of splitting a deceased donor liver cannot be transferred to the German situation because most organs are marginal donors anyway and the vast majority of left lateral lobes of organs which are suitable for a splitting procedure are offered for pediatric recipients. This is the reason why we decided to offer potential recipients with colorectal liver metastases the option of living donor liver transplantation. The advantages are obvious: we look at an expansion of liver transplantation indications without debiting the deceased donor pool; the risk for the donor is maximally reduced since we only use left lateral lobes and the risk for the recipient is also relatively low since we can plan the finalization of the hepatectomy according to the liver function capacity. Although the option of AB0-incompatible would be feasible in living donation, we preclude this in our actual protocol since this immunological challenge is too dangerous at this stage.

In summary, we present a study design which focus on strict patient’s selection. This is in our opinion the key for favorable results in our transplantation approach to achieve an excellent long-term outcome.

## Conclusions

With the proposed study protocol, it might be possible to cure well-selected patients with advanced colorectal cancer. However, due to the organ shortage, we decided to use left lateral split organs from healthy living donors with the minimal possible risk for the donor. This innovative approach might be an additional tool in surgical oncology.

## Additional file

Additional file 1: SPIRIT checklist within the LIVER-T(W)O-HEAL study. (DOC 121 kb)
